# Alternative Imaging Modalities in Ischemic Heart Failure (AIMI-HF) IMAGE HF Project I-A: study protocol for a randomized controlled trial

**DOI:** 10.1186/1745-6215-14-218

**Published:** 2013-07-16

**Authors:** Eileen O’Meara, Lisa M Mielniczuk, George A Wells, Robert A deKemp, Ran Klein, Doug Coyle, Brian Mc Ardle, Ian Paterson, James A White, Malcolm Arnold, Matthias G Friedrich, Eric Larose, Alexander Dick, Benjamin Chow, Carole Dennie, Haissam Haddad, Terrence Ruddy, Heikki Ukkonen, Gerald Wisenberg, Bernard Cantin, Philippe Pibarot, Michael Freeman, Eric Turcotte, Kim Connelly, James Clarke, Kathryn Williams, Normand Racine, Linda Garrard, Jean-Claude Tardif, Jean DaSilva, Juhani Knuuti, Rob Beanlands

**Affiliations:** 1 Division of Cardiology, (including Cardiac Imaging, The National Cardiac PET Centre, The Heart Failure Program, and the Cardiac Research Methods Centre), Department of Medicine, University of Ottawa Heart Institute, 40 Ruskin Ave, Ottawa, ON K1Y 4W7, Canada; 2 University of Ottawa, 75 Laurier Avenue East, Ottawa, ON K1N 6N5, Canada; 3 Department of Radiology, The Ottawa Hospital, Module S, 501 Smyth Road, Ottawa, ON K1H 8L6, Canada; 4 Montreal Heart Institute, 5000 Bélanger Street, Montréal, QC H 1T 1C8, Canada; 5 London Health Sciences Centre, 800 Commissioners Road East, PO Box 5010, London, ON N6A 5W9, Canada; 6 Turku PET Centre, c/o Turku University Hospital, P.O. Box 5220521, Turku, Finland; 7 University of Alberta, 116 St, Edmonton, AB T6G 2R3, Canada; 8 Université de Québec, 2325 Rue de l'Université, Québec City, QC, Canada; 9 St. Michael's Hospital, 30 Bond St, Toronto, ON M5B 1W8, Canada; 10 Université de Sherbrooke, 2500 boul. de l'Université, Sherbrooke, QC J1K 2R1, Canada; 11 Sunnybrook Health Sciences Centre, 2075 Bayview Ave, Toronto, ON M4N 3M5, Canada; 12 Dalhousie University, 1236 Henry St, Halifax, NS B3H 1B6, Canada

## Abstract

**Background:**

Ischemic heart disease (IHD) is the most common cause of heart failure (HF); however, the role of revascularization in these patients is still unclear. Consensus on proper use of cardiac imaging to help determine which candidates should be considered for revascularization has been hindered by the absence of clinical studies that objectively and prospectively compare the prognostic information of each test obtained using both standard and advanced imaging.

**Methods/Design:**

This paper describes the design and methods to be used in the Alternative Imaging Modalities in Ischemic Heart Failure (AIMI-HF) multi-center trial. The primary objective is to compare the effect of HF imaging strategies on the composite clinical endpoint of cardiac death, myocardial infarction (MI), cardiac arrest and re-hospitalization for cardiac causes.

In AIMI-HF, patients with HF of ischemic etiology (n = 1,261) will follow HF imaging strategy algorithms according to the question(s) asked by the physicians (for example, Is there ischemia and/or viability?), in agreement with local practices. Patients will be randomized to either standard (SPECT, Single photon emission computed tomography) imaging modalities for ischemia and/or viability or advanced imaging modalities: cardiac magnetic resonance imaging (CMR) or positron emission tomography (PET). In addition, eligible and consenting patients who could not be randomized, but were allocated to standard or advanced imaging based on clinical decisions, will be included in a registry.

**Discussion:**

AIMI-HF will be the largest randomized trial evaluating the role of standard and advanced imaging modalities in the management of ischemic cardiomyopathy and heart failure. This trial will complement the results of the Surgical Treatment for Ischemic Heart Failure (STICH) viability substudy and the PET and Recovery Following Revascularization (PARR-2) trial. The results will provide policy makers with data to support (or not) further investment in and wider dissemination of alternative ‘advanced’ imaging technologies.

**Trial registration:**

NCT01288560

## Background

The multifaceted Canada-Finland collaborative research program, Imaging Modalities to Assist with Guiding Therapy and the Evaluation of Patients with Heart Failure (IMAGE-HF), is designed with the following overall objectives: 1) to determine the impact of emerging imaging strategies on relevant clinical outcomes and decision making in patients with HF; 2) to establish standardized quality assurance (QA) measures and central databases in order to achieve reliable outcome driven research; and 3) to apply this as a platform for evaluation of new and emerging imaging and serum biomarkers in HF. The program consists of three separate randomized controlled trials. Project 1A is designed to compare the effect of HF imaging strategies in the evaluation and diagnosis of ischemic heart disease (IHD). Project 1B evaluates the utility of cardiac magnetic resonance (CMR) imaging in addition to standard echocardiography in the evaluation and diagnosis of non-ischemic HF (with either preserved or reduced ejection fraction) in comparison to the standard echocardiography alone. Project 1C is comparing two imaging modalities for the detection of coronary artery disease (standard coronary angiography versus cardiac computerized tomography scans).

### Project 1A is the focus of this publication

Despite multiple advances in cardiovascular disease, the morbidity and mortality of patients with heart failure (HF) in the setting of IHD remains high. Although it is believed that most patients with symptoms of significant ischemia may benefit from revascularization, decisions regarding revascularization in those with advanced ventricular dysfunction and no significant ischemia are complex, and the applicability of current clinical trial data is often challenged by limited patient selection. Over the past three decades, information describing cardiac structure, function, perfusion, hemodynamics, and metabolism obtained from noninvasive cardiac imaging studies has been used to guide management decisions for patients with HF. Although this anatomic and physiologic information adds value to clinical care, an accepted strategy is still debated regarding the optimal testing sequence approach to efficiently identify the treatment strategy most likely to improve outcomes. Consensus on proper use of cardiac imaging studies has been hindered by absence of clinical studies that objectively compare the independent treatment-related prognostic information of each test obtained using standardized methods. Uniformity of reporting formats also needs to be improved in order to provide a clearer working scheme for clinicians.

Alternative Imaging Modalities in Ischemic Heart Failure (AIMI-HF) (Project I-A of the Imaging Modalities to Assist with Guiding Therapy and the Evaluation of Patients with Heart Failure, IMAGE-HF program) is a multicenter trial with the primary objective of comparing the effect of HF imaging strategies on the composite clinical endpoint of cardiac death, myocardial infarction (MI), resuscitated cardiac arrest and cardiac re-hospitalization (worsening heart failure, acute coronary syndrome, arrhythmia). Patients with an ischemic heart disease (IHD) etiology will follow HF imaging strategy algorithms according to the question(s) asked by the physicians (for example, is there ischemia and/or viability?), in agreement with their local clinical practices for standard and alternative imaging. Patients will be randomized to either standard imaging modalities for ischemia and/or viability (SPECT) or advanced imaging modalities, namely cardiac magnetic resonance imaging (CMR) or positron emission tomography (PET). Secondary objectives include the effect of HF imaging strategies on the incidence of revascularization procedures, left ventricular remodeling, HF symptoms and quality of life, as well as a health economic evaluation. A biomarker substudy (on renal function, left ventricular remodeling and a selected set of biomarkers), assessing mechanisms underlying specific cardiovascular events, is also planned (see Additional file 1).

#### Coronary revascularization and ischemic heart failure

Among patients with coronary artery disease (CAD) and heart failure, mortality rates range from 10 to 60% at 1 year [1-11]. CAD is the most common cause of HF, however the role of revascularization in these patients is often unclear. Significant concerns remain about perioperative morbidity and mortality [8,10-14]. The recent Surgical Treatment for Ischemic Heart Failure (STICH) trial [15] did not demonstrate a significant benefit for coronary artery bypass graphing (CABG) surgery compared to medical therapy, for the primary endpoint of all-cause mortality in patients with LV dysfunction (ejection fraction {“EF”} ≤ 35%) and coronary disease eligible for CABG; although there was benefit for secondary endpoints of cardiovascular death and cardiovascular endpoints. STICH focused on IHD rather than on chronic HF with systolic dysfunction, and the outcomes of the many patients who were screened but did not undergo revascularization remain unknown [16]. Unfortunately, the STICH trial has not provided the final answer on the role of revascularization for patients with chronic HF; nor did it evaluate the role of advanced imaging in decision making for revascularization in this patient population.

#### Imaging in ischemic heart failure

Increasingly over the past three decades, information describing cardiac structure, function, perfusion, hemodynamics, and metabolism obtained from noninvasive cardiac imaging studies has been used to guide management decisions for patients with HF. Although this anatomic and physiologic information adds value to clinical care, an accepted imaging strategy has not evolved that tailors the testing sequence to specific presenting features of individual patients to efficiently identify the treatment strategy most likely to improve outcomes. Consensus on proper use of cardiac imaging studies has been hindered by the absence of clinical studies that objectively compare the independent treatment-related prognostic information of each test obtained using standardized methods.

Observational data has demonstrated that methods to define ischemia, viability and scar can identify high risk patients likely to benefit (or not) from revascularization [17-22].

The long-term impact of newer or alternative imaging strategies used for the revascularization decision processes has not been evaluated prospectively in HF. Revascularization has the potential to restore function to dysfunctional viable myocardium but not scar. Our group and others have shown that patients with dysfunctional but viable hibernating myocardium are at high risk for cardiac events if they do not undergo timely revascularization [20,23].

Until recently, data from predominantly observational studies had shown that when viability is present, patients have better outcomes with revascularization [24-26]. The PET and Recovery Following Revascularization (PARR-2) trial [27] represents the largest randomized study to evaluate viability imaging in patients with severe LV dysfunction. Overall, there was a trend for benefit for Fluorodeoxyglucose (^18^F) positron emission tomgoraphy scan (FDG PET) assisted management over standard care. When the adherence to imaging recommendations was considered, there was a significant outcome benefit. A high risk subgroup demonstrated a significant mortality benefit [20,27]. Recently, in a post-hoc analysis a significant reduction in events was observed in a subset of patients at the Ottawa site (Ottawa-FIVE) [28]. The results suggest outcome benefits can be achieved using FDG PET in an experienced center with ready access to FDG and interactions with HF and revascularization teams.

Although PARR2 was unique as a randomized controlled trial for imaging viability, it was underpowered for the primary outcome. Larger prospective randomized studies are needed, although undertaking such studies can be challenging [16,29,30]. The STICH viability study [29] did not include a comparison to late gadolinium enhanced CMR, other modalities, nor evaluate the role of stress induced ischemia. Finally, although care was taken to standardize imaging acquisition and transfer of data, standardization was not as rigorous as has recently been achieved in the CADRE Ontario provincial registry [31].

Results from the STICH Viability substudy [29] suggest that identification of viable myocardium by single photon emission computed tomography (SPECT) or dobutamine stress echocardiography (DSE) do not add value in patients selected for surgical revascularization. The STICH Viability substudy results may be explained by a patient population that was at lower risk, with patients already acceptable for revascularization, having more single vessel disease, infrequent previous CABG, low incidence of renal dysfunction and predominantly without heart failure. For such patients, it may be argued that viability imaging is not needed. This is in contrast to sicker populations in studies such as PARR2 [20,27], where physicians were uncertain about revascularization decisions and, therefore, needed viability assessment. Viability testing was not randomized in STICH [15,16,29]. The authors acknowledged the potential for selection bias [29]. Furthermore, only 19% of patients in the substudy were considered to have nonviable myocardium, which is far less than in most previous studies [29,32,33]. Analyses combining DSE and SPECT results were performed. Ischemia and hibernation imaging were not reported. More advanced (or alternative) ischemia and viability imaging modalities (that is, using PET and CMR) were not evaluated.

Thus the STICH results need to be interpreted cautiously [16,30], and the limitations along with the other observational and randomized data, justify the need for a prospective randomized trial to evaluate imaging strategies in patients with heart failure.

AIMI-HF is a large randomized controlled trial, comparing ‘advanced imaging technologies’ (PET and CMR) to standard imaging (SPECT). The findings will provide policy makers with data to support (or not) further investment in and dissemination of alternative or advanced technologies.

### Study hypotheses and objectives

#### Primary hypothesis

In patients with HF due to IHD with left ventricular ejection fraction (LVEF) ≤ 45%, a management algorithm that applies alternative imaging strategies (PET or CMR) achieves a better clinical outcome measured as the composite clinical endpoint of cardiac death, MI, resuscitated cardiac arrest and cardiac re-hospitalization (hospitalization due to heart failure, acute coronary syndrome or arrhythmia) than an approach with standard care using SPECT imaging.

Secondary hypotheses

1) Compared to standard care, in patients with HF due to IHD with LVEF ≤45% a management algorithm that applies alternative imaging modalities (PET or CMR) achieves: a) more efficient use of revascularization procedures with similar complication rates than standard care imaging strategies; b) better HF and angina symptom reduction; c) better quality of life (QoL), measured using MLHFQ and EQ5D; and d) is cost-effective.

2) In patients with HF due to IHD with LVEF ≤45%, a HF management algorithm that applies PET achieves a better primary (composite clinical endpoint) and secondary outcomes (revascularizations, remodeling, QoL, cost effectiveness) compared to one that applies CMR.

#### Primary objective

The primary objective of AIMI-HF is to compare the effect of HF imaging strategies on the composite clinical endpoint of cardiac death, MI, resuscitated cardiac arrest and cardiac re-hospitalization (WHF, ACS, and arrhythmia). Patients with HF due to an ischemic heart disease (IHD) etiology of LV dysfunction will follow HF imaging strategy algorithms according to the question(s) asked by the physicians (Is there ischemia and/or viability?), in agreement with their local practices for standard and alternative imaging.

#### Secondary objectives

To compare the effect of *HF imaging strategies* on:

1. The incidence of revascularization procedures (percutaneous coronary intervention {PCI}, CABG).

2. LVEF,

3. HF symptoms and New York Heart Association Functional Class (NYHA) class.

4. QOL (Minnesota Living with Heart Failure questionnaire (MLHFQ), the EQ5D).

5. Health economics. Costs will be estimated through regression analysis and cost effectiveness will be assessed through decision modeling.

6. The safety of imaging tests measured by cumulative radiation, adverse reactions to imaging contrast agents and stress testing agents will also be determined.

## Methods/Design

The AIMI-HF is a randomized controlled trial to compare the effectiveness of HF imaging strategies in patients with HF due to IHD. Patients enrolled will have LV systolic dysfunction due to IHD where evaluation of ischemia and viability is relevant. Patients will be allocated in a concealed fashion to standard (SPECT) versus advanced (PET or CMR) imaging. In addition, a registry will be maintained of patients undergoing standard or advanced imaging based on clinical decisions.

A survey sent to participating centers revealed that most of them could not provide stress echocardiography consistently within the requested timeframe as per the research protocol. This was especially true for dobutamine stress-echo (DSE) and viability protocols. The decision not to include this method was mainly due to statistical considerations; since if only a few centers elected to use this standard method, there may not be enough patients to adequately compare it with other methods (and site bias might also be involved).

### Study participants

Patients with clinical HF (see Table 1 for definition) or severe LV systolic dysfunction who need further definition of ischemia, viability or scar and meet the following inclusion and exclusion criteria, will be considered for entry into this trial. Patients will be prospectively randomized to standard (SPECT) versus advanced (PET or CMR) imaging. Patients who meet inclusion criteria but cannot be randomized due to clinical management decisions, yet undergo standard or advanced imaging, will be entered into a registry.

**Table 1:** Criteria for the clinical diagnosis of heart failure

**At least one of each of the following symptoms and signs in the last 12 months:**^**a**^	
**Symptoms**	**Signs**
▪ Paroxysmal nocturnal dyspnea	▪ Pulmonary rales (post cough)
▪ Orthopnea	▪ Jugular venous pressure (JVP) ≥5 cm above sternal angle
▪ Dyspnea upon mild or moderate exertion	▪ Lower extremity oedema
	▪ Chest x-ray demonstrating pleural effusion, pulmonary congestion, or cardiomegaly

^a^If LVEF is 30% or less, no signs or symptoms of HF are required for eligibility.

Inclusion criteria:

• Age >18 years

and

• Known or highly suspected coronary artery disease (CAD) documented by coronary angiography or by history of previous MI or evidence of moderate ischemia or scar based on prior imaging.

and

• LV dysfunction most likely attributable to ischemic heart disease with EF ≤45% measured by any acceptable means (echo, nuclear RNA, PET or SPECT perfusion, angiography, CMR) within the previous 6 months and NYHA class II to IV symptoms within the past 12 months or

• LV dysfunction most likely attributable to ischemic heart disease with EF ≤30% measured by any acceptable means (echo, nuclear RNA, PET or SPECT perfusion, angiography, CMR) within the previous 6 months AND NYHA class I within the past 12 months.

Exclusion criteria:

• Severe medical conditions that significantly affect the patient's recommended management (for example, severe COPD, active metastatic malignancy) and would preclude revascularization.

• <4 weeks post-ST segment elevation myocardial infarction (STEMI)

• Already identified as not suitable for revascularization

• Emergency revascularization indicated

• Severe valvular heart disease requiring valve surgery

• Pregnancy, breast feeding

• Potential for non-compliance to tests involved in this protocol

• Incapacity to provide informed consent

### Randomization

**Figure 1:**
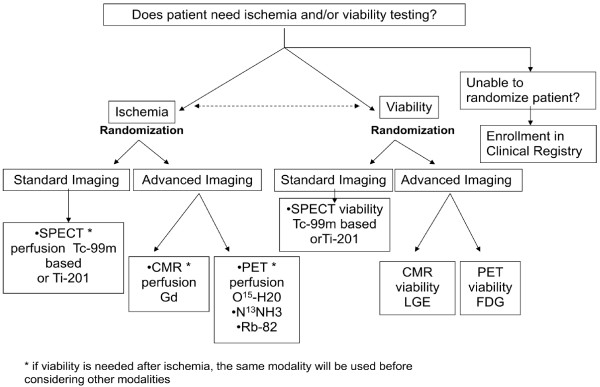
Overall randomization scheme for patients enrolled in Alternative Imaging Modalities in Ischemic Heart Failure (AIMI-HF).

Patients will be randomized according to a pre-defined randomization scheme and availability of imaging procedures at individual participating centers. All eligible patients will be randomized to either standard or advanced imaging modalities for ischemia and/or viability testing. Participating sites with the capability for two advanced imaging modalities will then be further randomized between each modality. If randomization is not possible due to local site factors, the patient can be entered into the registry (Figure 1). The ratio of advanced to standard imaging will be 2:1.

### Blinding

The study is not blinded given the nature and purpose of the interventions. Knowledge of imaging results and potential gained from the intervention will need to be considered to implement the appropriate treatment strategy. Therefore, performance bias (that is, systematic differences between groups in the care provided or exposure to factors other than the interventions of interest) and attrition bias (that is, systematic differences between groups in withdrawals from the study) may occur. Detection bias is still a potential concern, but an independent assessor will evaluate the objectively defined primary outcome and an adjudication committee will independently review and adjudicate each clinical event blinded to treatment randomization.

### Measurements

Standard imaging protocols have been defined by the IMAGE-HF Standardization team, using nationally recognized protocols (Additional file 2) GFR will be estimated (eGFR) using the Modified Diet in Renal Disease (MDRD) equations based on current recommendations [34-37]. CBC, electrolytes, urea, creatinine will be measured locally on randomization. This creatinine measurement will serve for local eGFR assessment (to ensure CMR eligibility). Further laboratory analyses will be collected and stored for future biomarkers analyses (at baseline and at 1 year in a subgroup of patients; Additional file 1).

### Subject evaluation

At baseline, demographic and clinical data will be collected from all participants on standardized case report forms. These data will be collected from the most recent, routine history and physical examination that has been completed by the treating physician. Quality of life questionnaires (EuroQol and Minnesota Living with Heart Failure) will be administered. In addition, the above-mentioned laboratory samples will be collected. To address a secondary objective, whenever possible, an echocardiogram will be performed if LV ejection fraction has not been determined by echocardiography within 6 months prior to randomization. As much as possible, imaging procedures will be performed within 4 weeks after entry into the trial. Subsequent telephone follow-up and repeat blood work will be performed on a predetermined schedule (Table 2). If the patient cannot be reached by telephone for their final assessment, a query will be made at government or national health care resources to verify if any corresponding events have occurred since the last visit (search of corresponding codes for cardiac death, MI, cardiac arrest and cardiac re-hospitalization for WHF, ACS, or arrhythmia). A follow-up echocardiogram will be requested at one year (LV remodeling, LVEF). Within 3 months of the baseline scan the treating physician will be asked to record the HF management plan.

**Table 2:** Patient assessment schedule

	**Baseline**	**3 months**	**6 months**	**12 months**	**18 months**	**24 months**	**36 months**	**48 months**
Imaging (SPECT, PET or CMR)	x							
Blood work	x			x				
Demographic and history	x							
Clinical data collected	x			x				
EuroQol questionnaire	x	x	x	x	x	x	x	x
MLWHF questionnaire	x	x	x	x	x	x	x	x
Telephone follow-up		x	x	x	x	x	x	x

*CMR* cardiac magnetic resonance, *MLWHF* Minnesota Living with Heart Failure, *PET* positron emission tomography, *SPECT* single photon emission computed tomography.

### Safety and ethics

This study was approved by the University of Ottawa Heart Institute Human Research Ethics Board, protocol #2010620-01H. In addition, before study initiation at each site, this protocol, and the informed consent form, as well as any advertisement for subject recruitment, was submitted for review and approval by each local participating site’s ethics committee charged with this responsibility and will be so submitted for future sites. These ethic committees will submit written notification of the approval to the investigator. This study will be conducted according to the Declaration of Helsinki, Good Clinical Practice and the TriCouncil Policy.

### Registry

Eligible and consenting patients that could not be randomized but undergo standard or advanced imaging based on clinical decisions, will be included in a registry. Measurement, subject evaluation and safety and ethics as outlined in the previous sections will apply to these registry patients. The registry patients for advanced imaging will be considered in the sample size calculation and data analysis (see below); however, the small number of patients expected to be part of the SPECT registry will not be considered in the primary analysis.

### Sample size

For the sample size determination, the estimated occurrence over one year of the composite clinical endpoint of cardiac death, MI, resuscitated cardiac arrest and cardiac re-hospitalization (WHF, ACS, arrhythmia) for PET is 27% and for standard care is 40%, based on the Ottawa-FIVE substudy [28] of the PARR-2 [27] study in which the composite event rates were 19% and 41%, respectively. These estimates were considered reasonable as it reflects the outcome rates that may be achievable at a facility with expertise and access to FDG PET imaging. There are no similar data upon which to draw an estimate for CMR. Considering the Schinkel *et al*. publication [38], which noted sensitivity of CMR to be between PET and standard care modalities and based on expert consensus from our IMAGE-HF workshop (16 December 2008 in Toronto, Canada), we estimate the event rate for CMR directed care would lie between the above values at 34%. Hence, an overall rate for the alternative (PET, CMR) modalities would be approximately 30%. The rates are considered conservative since the mean duration of follow-up will be 2 years, whereas the PARR-2 study was 1 year.

For the primary hypothesis, using the two-sided log-rank test for comparing advanced (PET + CMR) versus standard modalities with a 2:1 patient allocation, a sample size of 495 patients (of which 330 are allocated to the advance modality and 165 are allocated to the standard modality) would be needed in order to detect a difference after 1 year in the composite clinical endpoint of 30% for the advanced modalities (PET, CMR) compared to 40% for standard care modalities (SPECT). This is calculated with a level of significance of 0.05 and power of 80%, and assuming a uniform accrual of patients over the 2.5 year recruitment period, a 1 year minimum follow-up period and a 10% loss from each study group. The difference of 10% was considered to be a minimal clinically important difference based on a consensus of the IMAGE-HF investigators.

For the secondary hypothesis, using the Cox regression model for comparing the advanced modalities PET versus CMR with an approximate 1:1 patient allocation, a sample size of 548 patients per modality is needed considering an anticipated rate for the composite clinical endpoint of 30% after 1 year, a level of significance of 0.05, a power of 80% and a loss to follow-up of 10%. The sample size calculation is complicated by the fact that 766 patients (383 per group) from the registry (in which patients in the registries were clinically directed to PET or CMR) will be combined in the analysis with the 330 patients (165 per group) from the randomized part of the study (in which patients in the randomized study will be randomly allocated to PET versus CMR versus standard modality) for a total of 1096 patients (548 per group). For a sample of 1096 patients, a Cox regression of the log hazard ratio of the composite clinical endpoint on the group allocation variable (PET versus CMR) with a conservative standard deviation of 0.5 (based on an approximate equal allocation to PET and CMR), achieves 80% power at a 0.05 significance level to detect a 40% increase in the hazard ratio to1.4. This increase in the hazard ratio was deemed to be the minimal clinically important difference based on a consensus among the IMAGE-HF investigators. The sample size includes an adjustment to accommodate confounding by indication for allocation patients to PET versus CMR by incorporating a multiple regression of the group allocation variable on the other covariates in the Cox regression model; a conservative estimate of this confounding was considered by taking a value of 0.25 for the multiple correlation coefficient R for the relationship between the group allocation variable and the set of covariates identified.

The total sample size is thus 1,096 + 165 = 1,261.

### Statistical analysis

Descriptive statistics will be used to summarize the characteristics of the patients for each imaging technology on demographic, clinical and site-related factors, and differences between these groups will be reviewed for their clinical significance.

#### Analysis populations

For the purposes of data analysis, three study populations will be considered: Intent-to-treat (ITT) Population, As-Treated Population and Per-protocol Population. The ITT population will be used for the main analysis for all primary and secondary objectives, except for the safety analysis where the as-treated population will be used. As a secondary analysis, the analyses will be repeated for the as-treated and per-protocol populations.

#### Primary analysis (advanced versus standard imaging)

For the primary analysis, the time-to-event of the composite clinical endpoint of cardiac death, MI, arrest and cardiac re-hospitalization (WHF, ACS, arrhythmia) will be compared between the advanced modality (PET or CMR) to an approach with standard care using SPECT imaging using survival analysis. Kaplan-Meier survival curves of the primary endpoint will be compared between the advanced and standard modalities with the log-rank test. Potential confounding variables of the relationship between the imaging technologies and the primary endpoint will be assessed. In particular, propensity scores based on patient factors (for example, in/outpatient, NYHA class, HF duration, diabetes, atrial fibrillation) and site factors (for example, time-to-imaging, time-to-therapy) will be used in the analysis if necessary to adjust for potential differences. A Cox proportional hazard models will be used to assess the occurrence of the endpoints between the imaging technologies (model will include a group indicator variable) adjusting for any pertinent baseline differences identified. The proportional hazards assumption underlying the Cox model will be assessed.

#### Secondary outcomes

For the secondary outcomes PCI, CABG, HF symptoms and NYHA class, chi-square tests will be used to compare the advanced and standard imaging technologies; logistic regression analysis will be used for adjusting any pertinent baseline differences identified. For the secondary outcomes LVEF, MLHFQ and EQ5D, analysis of variance will be used to compare trends over time between the advanced and standard technologies. Analysis of covariance will be used for adjusting any pertinent baseline differences identified.

#### Economic evaluation

For secondary objective 5, a cost-effectiveness analysis of advanced versus standard modality groups will be conducted. Analysis will take the form of a cost-utility analysis with cost effectiveness assessed in terms of the incremental cost per quality life year. Analysis will incorporate data on resource use and patients utility values for the period from initiation of treatment to study termination. Resource use will be assessed through review of patient charts and patient utility values will be derived using the EQ5D and MLHF. A decision model will be created to estimate long-term costs and quality adjusted life years (QALYs) for all comparators. Uncertainty within the analysis will be assessed through Monte Carlo and other simulation techniques.

#### Safety analysis

For the secondary objective 6, safety will be evaluated by documenting all adverse events. Descriptive statistics (frequency distributions, numerical descriptors) and 95% CIs will be calculated. The as-treated population will be the main analysis population for this safety evaluation.

#### Secondary analysis (PET versus CMR)

For the secondary analysis, comparing the PET and CMR modalities, potential confounding variables of the relationship between the imaging technologies and the primary endpoint will be assessed. In particular, propensity scores based on patient factors (for example, in/outpatient, NYHA class, HF duration, diabetes, atrial fibrillation) and site factors (for example, time-to-imaging, time-to-therapy) will be used in the analysis if necessary to adjust for potential differences between PET and CMR. A Cox proportional hazard models will be used to assess the occurrence of the endpoints between the imaging technologies (the model will include a group indicator variable) adjusting for any pertinent baseline differences identified. The proportional hazards assumption underlying the Cox model will be assessed. The secondary outcomes will be analyzed in a similar fashion.

#### Missing data

‘Missingness’ is considered to be missing at random (MAR) and mixed methods repeated measures (MMRM) and multiple imputation techniques will be used for handling missing data. In particular, for continuous outcomes at multiple time points MMRM will be used.

### Study management

The IMAGE-HF trial is managed by an Executive Committee consisting of clinicians specialized in diagnostic imaging and/or heart failure and experts in biostatistics, physics and radiochemistry, as well as a larger Steering Committee consisting of members of the Executive Committee and representatives of all the initial study centers. (Table 3) In addition there is an events adjudication committee, which will independently review and adjudicate each clinical event blinded to treatment randomization. Since all the imaging approaches are part of standard clinical practice, no interim analysis is planned, but there will be independent data safety monitoring board (DSMB), which will review the safety data on a periodic basis; the frequency of the meetings and the charter governing the DSMB will be finalized at the first meeting of the DSMB.

**Table 3:** Imaging Modalities to Assist with Guiding Therapy and the Evaluation of Patients with Heart Failure (IMAGE-HP) participating

**IMAGE-HF Participating Sites**	
**Investigator**	**Role**
**University of Ottawa Heart Institute**	
R Beanlands	Co-Principal Investigator IMAGE-HF, Canada
G. A. Wells	Principal Investigator CRMC
R. deKemp	Principal Investigator QA Program
D. Birnie	Co-Principal Investigator Project IIA
L. Mielniczuk	Co-Principal Investigator Project IA
K. Chan	Site Principal Investigator
B. Chow	Principal Investigator Project IC
L. Garrard	Project Management
R. Hessian	Investigator
T. Ruddy	Investigator
RA Davies	Investigator
H. Haddad	Investigator
A. Dick	Investigator
C. Dennie	Investigator
D. Coyle	Investigator
B. McArdle	Investigator
T. Dowsley	Investigator
G. Dwivedi	Investigator
J. DaSilva	Investigator
C. Kelly	Research Coordinator
E. Moga	Research Coordinator
R. Klein	Core Lab Manager
K. Williams	Statistician
R. Fleming	Research Coordinator
M. Boomgaardt	Research Coordinator
**Montreal Heart Institute-Université de Montréal**	
JC Tardif	Investigator
E. O'Meara	Co-Principal Investigator Project IA
M. Friedrich	Investigator
J. Rouleau	Investigator
T. Heinonen	Investigator
F. Marcotte	Investigator
N. Racine	Investigator
H. Q Ly	Investigator
J. Morrissette	Research Coordinator
H. Brown	Research Coordinator
**University of Alberta**	
I. Paterson	Principal Investigator Project IB
L. Lalonde	Investigator
J. Ezekowitz	Investigator
M. Irwin	Research Coordinator
**University of Turku**	
J. Knuuti	Co-Principal Investigator IMAGE-HF, Finland
H. Ukkonen	Investigator
S. Yla-Herttuala	Investigator
H. Leskinen	Investigator
A. Saraste	Investigator
T. Vasankari	Research Coordinator
K. Lahtonen	Research Coordinator
M. Tarkia	Site Project Manager
**University Central Hospital, Helsinki**	
M. Laine	Site Principal Investigator
H. Hanninen	Investigator
M. Pietila	Research Coordinator
**University of Kuopio**	
J. Hartikainen	Site Principal Investigator
S. Karkkainen	Investigator
I. Kaivonurmi	Research Coordinator
M. Sutinen	Research Coordinator
**Sunnybrook Health Sciences Centre**	
G. Wright	Site Co-Principal Investigator
K. Connelly	Site Co-Principal Investigator
R. Myers	Investigator
C. Cunningham	Investigator
E. Crystal	Investigator
A. Leber	Investigator
M. Mohammed	Research Coordinator
J. Malko	Research Coordinator
**University of Calgary**	
A. Howarth	Site Co-Principal Investigator
T. Anderson	Site Co-Principal Investigator
A. Krysk	Investigator
S. Hutchison	Investigator
N. Merchant	Investigator
S. Weeks	Investigator
R. Sandonato	Research Coordinator
S. Rivest	Research Coordinator
J. Veenhuyzen	Research Coordinator
M. Seib	Research Coordinator
B. Madden	Research Coordinator
D. Durand	Research Coordinator
**London Health Sciences**	
M. Arnold	Site Principal Investigator
G. Wisenberg	Investigator
T. Lee	Investigator
F. Prato	Investigator
J. White	Co-Principal Investigator Project IIA
K. Carter	Research Coordinator
**Laval University**	
E. Larose	Site Principal Investigator
P. Pibarot	Investigator
B. Cantin	Investigator
J. Carange	Research Coordinator
K. Bibeau	Research Coordinator
**St. Michael's Hospital**	
M. Freeman	Site Co-Principal Investigator
K. Connelly	Site Co-Principal Investigator
H. Leong-Poi	Investigator
G. Moe	Investigator
A. Al-Hesayen	Investigator
J. Sloninko	Research Coordinator
**Hamilton**	
V. Tandon	Site Principal Investigator
K. Gulenchyn	Investigator
F. Spence	Investigator
A. Khoorshed	Research Coordinator
**Sherbrooke**	
E. Turcotte	Site Principal Investigator
S. Lepage	Investigator
Paul Farand	Investigator
S. Joncas	Resident, recruitment
E. Lavallee	Research Coordinator
**Halifax**	
M. Rajda	Site Principal Investigator
R. Stewart	Investigator
J. Clarke	Investigator
S. Burrell	Investigator
B. Clarke	Investigator
S. Yarn	Research Coordinator
M. MacFarlane	Research Coordinator
**Winnipeg**	
M. Kass	Site Principal Investigator
J. Tan	Investigator
T. Moore	Research Coordinator
A. Munoz	Research Coordinator
**QA Core Labs**	
R. Klein	Core Lab Manager (Ottawa)
R. deKemp	PET, SPECT QA Core Lab Team Leader (Ottawa)
B. McArdle	PET, SPECT QA Core Lab (Ottawa)
J. Renaud	PET, SPECT QA Core Lab (Ottawa)
K. Chan	ECHO QA Core Lab Team Leader (Ottawa)
J. White	CMR QA Core Lab 1A Team Leader (London)
I. Pauchard	CMR QA Core Lab 1A (London)
I. Patterson	CMR QA Core Lab 1B Team Leader (Edmonton)
P. L’Allier	ICA QA Core Lab Team Leader (Montreal)
B. Chow	CTA QA Core Lab Team Leader (Ottawa)
**Steering Committee**	
R. Beanlands	
G. A. Wells	
J. Knuuti	
M. Friedrich	
G. Wright	
M. Arnold	
J.C. Tardif	
P. Pibarot	
S. Ylä-Herttuala	
R. deKemp	
**DSMB**	
A. Krahn, Chair	
J. Fallavollita	
L. Thabane	
**Events**	
H. Haddad, Chair	
D.S. Beanlands	
L. Duchesne	
J. Ezekowitz	
R. A. Davies	

Blood samples for the biomarkers ancillary study will be stored at the Montreal Heart Institute central laboratory for analyses to be performed after study completion.

## Trial status

At the time of this manuscript preparation, the IMAGE IA trial is currently in the second year of active enrollment. We have enrolled a total of 249 patients, representing 20% of anticipated total enrollment. The study is active in a total of 13 sites across Canada and Finland. We anticipate study completion of enrollment by December 2015.

## Abbreviations

AIMI-HF: Alternative Imaging Modalities in Ischemic Heart Failure; CABG: Coronary artery bypass graphing; CAD: Coronary artery disease; CKD: Chronic kidney disease; CMR: Cardiac magnetic resonance imaging; DSE: Dobutamine stress echocardiography; DSMB: Data safety monitoring board; EF: Ejection fraction; eGFR: Estimated glomerular filtration rate; FDG PET: Fluorodeoxyglucose (^18^F) positron emission tomgoraphy scan; HF: Heart failure; IHD: Ischemic heart disease; IMAGE-HF: Imaging Modalities to Assist with Guiding Therapy and the Evaluation of Patients with Heart Failure; ITT: Intent-to-treat; JVP: Jugular venous pressure; LVEF: Left ventricular ejection fraction; MAR: Missing at random; MDRD: Modification of Diet in Renal Disease; MI: Myocardial infarction; MLWHF: Minnesota Living with Heart Failure; MMRM: Mixed methods repeated measures; NYHA: New York Heart Assocation Functional Class; PARR-2: PET and Recovery Following Revascularization; PCI: Percutaneous coronary interventsion; PET: Positron emission tomography; QA: Quality assurance; QALYS: Quality adjusted life years; SOPs: Standard operating procedures; SPECT: Single photon emission computed tomography; STEM: Segment elevation myocardial infarction; STICH: Surgical treatment for ischemic heart failure.

## Competing interests

The following authors have competing interests to disclose; R Beanlands is a consultant for Lantheus Medical Imaging, DraxImage; and has research funding from Lantheus Medical Imaging, GE, and MDS Nordion E O’Meara has research funding from Johnson & Johnson for the Cardiorenal-anemia syndrome in HF. R deKemp is a consultant for Jubilant DraxImage; and has research funding from the following: Lantheus Medical Imaging, GE, MDS Nordion. He also receives revenues from rubidium generator technology licensed to Jubilant DraxImage, and receives revenues from FlowQuant software sales. R Klein is a consultant for Jubilant DraxImage. He receives revenues from rubidium generator technology licensed to Jubilant DraxImage and hereceives revenues from FlowQuant software sales. T Ruddy has Research funding from Nordion, Inc, GE Healthcare and Atreus, Inc. For all other authors, there are no competing interests.

## Authors’ contributions

RB, EO, LMM, GAW, JK conceived the study, participated in its design and coordination, and helped to draft the manuscript. EO and LMM are co-principle investigators of this project. LG is involved in the study design and project management and helped to draft the manuscript. RdK and RK established and will monitor the standardization of the imaging modalities. DC designed and will coordinate the economic evaluation. The following contributed to design of trial and will be involved with conducting the trial: BMcA, IP, JAW, MA, MF, PP, AD, EL, BC, CD, HH, TR, HU,GW, BC,MGF, ET, KC, JC, NR, JCT, JR. All authors read and approved the final manuscript.

## Supplementary Material

Additional file 1 Left ventricular remodeling and biomarkers ancillary study – a synopsis. Click here for file

Additional file 2 Standardization and quality assurance (IMAGE-QA). Click here for file
