# Supplementary material for: Alternative Imaging Modalities in Ischemic Heart Failure (AIMI-HF) IMAGE HF Project I-A: study protocol for a randomized controlled trial
Source: Trials. 2013 Jul 16;14:218. doi: 10.1186/1745-6215-14-218 (PMC3729711; doi:10.1186/1745-6215-14-218)
Supplement: Additional file 1 — Left ventricular remodeling and biomarkers ancillary study – a synopsis. [file 1745-6215-14-218-S1.doc]

Additional file 1: Left ventricular remodeling and biomarkers ancillary study – a synopsis

Left ventricular remodeling (and related biomarkers) is influenced both by appropriate choices of revascularization and by the presence of chronic kidney disease (CKD). This will be measured by echocardiography and serum fibrosis and collagen biomarkers in a subgroup of patients included in the AIMI-HF trial.

Patients with HF and CKD constitute a large subpopulation of HF patients at particularly high cardiovascular risk. Both cardiac imaging and revascularization are challenging in this group of patients, where there is an even clearer need to determine if the use of certain advanced or alternative cardiac imaging modalities can modify revascularization decisions and outcomes.

Risk stratification using serum and/or imaging biomarkers could also improve patient selection for angiography and revascularization procedures (whether kidney disease is present or not). In addition, selected biomarkers could be used to examine the mechanisms leading to increases in cardiovascular events in patients with CKD and HF.

In 6 participating centers (Montreal, Ottawa, Quebec, Edmonton, London, Sherbrooke) the ancillary study on chronic kidney disease, LV remodeling and biomarkers will be performed (on an estimated 300 patients). For patients participating in the substudy, the following biomarkers will be measured both at baseline and at 1 year: creatinine (as a central assessment, for central eGFR evolution assessment), PIIINP, NT-proBNP, hs-cTnT, hs-CRP, osteopontin will be measured at the MHI central laboratory. Also a complete echocardiography will be performed at baseline and at one year and the echocardiographic data will be used in combination with the biomarkers (as well as clinical and other imaging data collected in this trial) to examine the mechanisms leading to cardiovascular events in patients with HF with and without CKD.
