# Supplementary material for: Alternative Imaging Modalities in Ischemic Heart Failure (AIMI-HF) IMAGE HF Project I-A: study protocol for a randomized controlled trial
Source: Trials. 2013 Jul 16;14:218. doi: 10.1186/1745-6215-14-218 (PMC3729711; doi:10.1186/1745-6215-14-218)
Supplement: Additional file 2 — Standardization and quality assurance (IMAGE-QA). [file 1745-6215-14-218-S2.doc]

Additional file 2: Standardization and quality assurance (IMAGE-QA)

The IMAGE-HF QA program aims to standardize several important aspects of the proposed clinical imaging research:

1. defining best current imaging practice for standard-care tests
2. disseminating advanced imaging technology and standards
3. promoting structured reporting and comprehensive imaging QA
4. ensuring consistent interpretation and patient management recommendations

For the IMAGE-HF project 1A (AIMI-HF), this includes standard operating procedures (SOPs) for SPECT, PET and MRI stress-rest perfusion (ischemia) and viability imaging, as well as structured reporting elements and quality assurance review by QA-CORE labs (SOPs and CRFs are posted on the IMAGE-HF website).

Standard-care imaging protocols

SPECT Perfusion and Viability:

IMAGE-HF-1A SOP SPECT-perfusion-viability (SMH) 2010.pdf

Based on St. Michael’s Hospital 2010 procedure manual for Nuclear Cardiology studies, according to current ASNC guidelines. Perfusion protocols include the use of Tc-99 m-sestamibi, Tc-99 m-tetrofosmin, and Thallium-201 tracers, with adenosine, dipyridamole or dobutamine pharmacologic stress, or treadmill exercise stress according to the Bruce protocol. Viability imaging is performed using Tl-201 with a rest-redistribution protocol, rest or nitrate-enhanced Tc-99 m-tracer imaging.

Advanced imaging protocols

PET perfusion:

IMAGE-HF-1A SOP PET-perfusion (UOHI) may 2011.pdf

Based on University of Ottawa Heart Institute 2011 procedures and ASNC Guidelines for rest-stress PET perfusion imaging using Rb-82 or N-13-ammonia tracers, with dipyridamole or dobutamine pharmacologic stress.

PET viability:

IMAGE-HF-1A SOP PET-viability (UOHI) may 2011.pdf

Based on University of Ottawa Heart Institute 2011 procedures and ASNC Guidelines for myocardial viability (hibernation) PET imaging using F-18-FDG tracer.

MRI perfusion:

IMAGE-HF-1A SOP CMR-perfusion (CanSCMR) march 2010.pdf

Based on Canadian Society of Cardiac MR 2010 imaging guidelines, using gadolinium contrast enhanced imaging at rest and following adenosine stress.

MRI viability:

IMAGE-HF-1A SOP CMR-viability (CanSCMR) march 2010.pdf

Based on Canadian Society of Cardiac MR 2010 imaging guidelines, using late gadolinium enhancement (GLE) imaging at rest to identify myocardial fibrosis (scar).

Common structured reporting elements

Stress-rest perfusion (ischemia) imaging with SPECT, PET or MRI:

HF15-Stress Perfusion Report

The following common parameters are included on the interpretation CRFs: Modality, Stressor, Tracer, Symptoms, ECG findings, LV ejection fraction, Segmental rest and stress perfusion and wall-motion scores, location and severity of myocardial ischemia and infarction (scar). Clinical recommendation for revascularization, and confirmation that the recommendation was communicated to the referring physician are captured on CRF.

Viability (hibernation or scar) imaging with SPECT, PET or MRI:

HF12-FDGPET&SPECT viability, HF13-Viability Report CMR

The following common parameters are included on the interpretation CRFs: Modality, Tracer(s), LV ejection fraction, Segmental wall-motion, perfusion and viability or scar scores, location and severity of scar and/or hibernating myocardium. Clinical recommendation for revascularization, and confirmation that the recommendation was communicated to the referring physician are captured on CRF.

Quality assurance CORE lab reviews (QA-CORE)

A limited subset of scans (10%) are targeted for over-reading interpretation at an experienced site identified as the CORE lab for each imaging modality. The first 2 scans (and 5% of the subsequent scans) from each imaging modality at each recruiting site are transferred to the corresponding modality QA-CORE lab for clinical interpretation and comparison to the site interpretation for quality assurance. Disagreements in the overall interpretation of the extent of LV ischemia, scar, hibernation or clinical recommendation for revascularization are resolved by subsequent consensus review between the site and CORE labs, and recorded on the corresponding CRFs: *HF12-QA, HF13-QA, HF15-QA*

QA-CORE labs for SPECT and PET are established at the University of Ottawa Heart Institute, and the QA-CORE lab for MRI is at the University of Alberta.
